# The effect of counseling with cognitive behavior approach on self-esteem and body image in lactating mothers: randomized clinical trial

**DOI:** 10.1186/s40359-023-01363-4

**Published:** 2023-11-18

**Authors:** Nasrin Zamiri-Miandoab, Mojgan Mirghafourvand, Fatemeh Nemati, Mahin Kamalifard

**Affiliations:** 1 Department of midwifery, faculty member of Khoy, University of Medical Sciences, Khoy, Iran; 2 Social Determinants of Health Research Center, Faculty of Nursing and Midwifery, Tabriz University of Medical Sciences, Tabriz, Iran; 3 Faculty of Education and Psychology, Tabriz, Iran; 4 Department of midwifery, Faculty of Nursing and Midwifery, Tabriz University of Medical sciences, Tabriz, Iran

**Keywords:** Self-esteem, Body image, Cognitive behavior therapy, Lactating mother

## Abstract

**Background:**

Some of the women experience low self-esteem and negative body image in pregnancy and postpartum. These two factors along with other factors can reduce the rate of exclusive breastfeeding among women. Cognitive-behavior therapy (CBT) is one of the psychological approaches that is effective on the betterment of many of the psychological and personality disorders such as body image disorders as well as improvement of self-esteem. The aim of this randomized control trial is to recognize the effects of CBT during pregnancy period on self-esteem, body image (primary outcome) and exclusive breastfeeding (secondary outcome).

**Method:**

In this randomized controlled trial, 70 eligible pregnant women referring to health centers of Tabriz- Iran were assigned to two groups of 35 as intervention and control groups using randomized blocking method. For intervention group, 8 sessions of CBT based on Cash and Strachan’s body image protocol and Michael Freeʼs for self-esteem were performed. Control group was received routine pregnancy care by their health provider. Rosenberg self-esteem scale and multidimensional body self-relation questionnaire (MBSRQ) were completed before intervention, immediately after intervention and 4 weeks after delivery. Likewise, exclusive breastfeeding questionnaire was completed 4 weeks after childbirth. Independent t-test, chi square and repeated measures ANOVA tests were used to analyze the data.

**Results:**

According to repeated measures ANOVA test and with controlling baseline score, the mean scores on self-esteem (AMD): 7.18; 95%confidence interval (CI): 4.43 to 9.94; p < 0.001)) and body image (AMD: 49.74; 95%CI = 28.57 to 70.91; p < 0.001) in the intervention group were significantly higher than the control group. Also, after intervention, the mean score of body image subscales including appearance evaluation (p = 0.010), appearance orientation (p = 0.001), fitness evaluation (p = 0.004), fitness orientation (p = 0.001), health evaluation (p = 0.001), health orientation (p = 0.018), and illness orientation (p = 0.002) was significantly higher in the intervention group than the control group.

**Conclusions:**

CBT was effective on the improvement of self-esteem and body image and through which might lead to the increase of exclusive breastfeeding among women.

**Trial registration number:**

IRCT20110524006582N33. First Date of registration: 17/10/2022. Submission ID 4ca86cd4-8459-4b86-9fe5-63f6a8184956.

## Back ground

Self-esteem, also known as self-respect, is a psychological concept that describes a positive or negative attitude toward oneself [1, 2]. Rosenberg believes that a person with high self-esteem recognizes and respects himself/herself as a valuable person [3]. Self-esteem is so significant that it has its own category in Maslow’s hierarchy [4]. A lack of self-esteem causes a slew of issues in people’s social relationships [5]. It also causes feelings of isolation and guilt, as well as sexual dysfunction, eating disorders [6], anxiety [7], and depression [8]. Throughout one’s life, one’s self-esteem can fluctuate [7]. Due to the physiological, anatomical, and psychological changes that women go through during pregnancy, it is one of the times when they experience low self-esteem [9]. Lack of self-esteem is associated with poor mental and physical health in mothers, and it has a negative impact on infant health as well as mother–infant attachment [10, 11].

Many factors influence self-esteem formation, including genetics, age, socioeconomic status, thought patterns [12], health conditions, parents, childhood events, and so on [7]. Similarly, social norms about the body, particularly the ideas of family members and friends, as well as deficiencies in appearance, can have a significant impact on self-esteem [13, 14]. Body image is another important and influential factor in self-esteem. Some studies show that poor body image is caused by low self-esteem, while others show that low self-esteem is caused by dissatisfaction with one’s body image [15].

Body image refers to a person’s beliefs and feelings about his/her physical appearance (such as height, weight, and body shape) and sexual attractiveness in comparison to society’s standards [16]. Although body image is an internal feeling, external factors such as friends, family members, the social environment, and the media all play important roles in shaping a person’s perception and feelings about his or her appearance [17]. According to studies, body image dissatisfaction among Iranian women has increased noticeably in recent years [18]. Body image, like self-esteem, changes throughout a woman’s life, including menstruation, pregnancy [19, 20], breastfeeding and postpartum [21]. Poor body image causes feelings of unattractiveness and dissatisfaction with marital relationships [22] as well as depression [23], eating disorders and low self-esteem [24]. Postpartum depression is more common in women who have a negative body image [25]. Similarly, mothers who were dissatisfied with their postpartum body image had a more negative attitude toward breastfeeding [26].

Self-esteem [27] and body image [28] are two of the most important and influential factors influencing exclusive breastfeeding. Currently, only 40% of infants worldwide receive exclusive breastfeeding. This is despite the fact that, according to UNICEF, if exclusive breastfeeding rates rise, more than 80,000 infants under the age of five will be saved from death [29]. It has been observed that when women are self-assured and properly supported by family members and others, they have a positive and long-term breastfeeding experience [30]. According to World Health Organization (WHO), starting and maintaining breastfeeding requires counseling as well as supportive and promotional programs [31].

Counseling is a process that aids in the improvement of an individual’s attitude, behavior, and personality. Furthermore, counseling improves communication skills, behavior change, mental health, and self-esteem empowerment [32]. CBT is a counseling technique that can be used alone or in conjunction with other approaches to treat personality disorders, mental disorders, depression, anxiety, and poor body image [33]. The therapist does not question the client’s feelings in this approach, but rather challenges the thoughts that cause such feelings and explains to the clients the process by which their thoughts lead to their feelings [34]. This method teaches mothers to avoid prejudgment and negative assessment and to demonstrate appropriate emotional responses in stressful situations such as pregnancy, breastfeeding, and postpartum periods [27]. By reviewing the literature, we came to the conclusion that CBT can probably be effective in increasing self-esteem and improving body image.Given some women’s low self-esteem and impaired body image, as well as the impact of these two factors on exclusive breastfeeding, we decided to investigate the effects of cognitive-behavioral pregnancy counseling on self-esteem, body image (primary outcomes), and exclusive breastfeeding (secondary outcome).

## Method

### Study design and participants

This randomized controlled trial was conducted on 70 pregnant women admitted to Tabriz health centers in 2020. The inclusion criteria for this study were a minimum of secondary education, a gestational age of 25–28 weeks, a first or second pregnancy, being a singleton, no diseases affecting the pregnancy process, not using drugs that affect mental-psychological problems, and no history of severe depression. Women who had a history of bleeding during the second stage of pregnancy, as well as a history of malignant breast diseases, were excluded from the study.

### Sampling

Following ethics committee approval and registration of the study in the Iranian Registry of Clinical Trials under the code IRCT20110524006582N33 in 2020/08/19, the sampling permission was obtained from the research deputy of the nursing-midwifery school and the deputy of the health department of East Azerbaijan province in Iran. The researcher went to Tabriz’s health centers in populous and socio-culturally diverse districts and extracted a list of pregnant women through the integrated health system (SIB system) in health centers; the women were then contacted via the phone number, and the research goals and methods were briefly explained to them over the phone. Moreover, they were analyzed based on inclusion and exclusion criteria, and if they were eligible and willing to participate in the study, they were invited to attend relevant centers at specific times. The goals and methods of the research were thoroughly explained during this face-to-face meeting, and if the pregnant mother was willing to participate in the study, written informed consent was obtained from her, and participants completed the Rosenberg self-esteem scale and the Multidimensional Body–Self Relations Questionnaire (MBSRQ) [35]. The study included women who scored less than 241 on the Multidimensional Body–Self Relations Questionnaire (MBSRQ) and less than 25 on the Rosenberg self-esteem scale [36–38]. Women with high scores on the questionnaire were excluded from the study. However, women who couldn’t participate regularly and women who had the mentioned exclusion criteria were excluded from study too. Participants who were entering the research were asked to fill out a socio-demographic questionnaire.

### Random allocation and intervention

Participants were randomly assigned to one of the two intervention and control groups (35 people in each group) using a stratified blocked randomization method (stratified by first or second pregnancy), with a block sizes of 4 and 6 and allocation ratio of 1:1. The allocation sequence was determined by a person not involved in the sampling and data collection. For allocation concealment, the type of intervention was written on paper and placed in opaque envelops numbered sequentially. The envelopes were opened in the order of the participants entering the study and the type of intervention received by each participant was determined.

Counseling based on a cognitive-behavioral approach was provided to the intervention group in 8 sessions of 60 to 90 min once a week by a master’s student of counseling in midwifery who had been trained for CBT by a professional psychologist. In addition, we have a psychologist on our team who supervised the intervention. The sessions were held in a calm environment in health centers considered for counseling. Body image counseling was based on Cash and Strachan’s body image workbook [39], while self-esteem counseling was based on Michael Free’s Cognitive Therapy in Groups: Guidelines and Resources for Practice [40]. Table 1 summarizes the content of the sessions. The Control group received routine pregnancy care from their health providers. At the end of the research, we provided brochures and pamphlets containing materials used in the intervention to the control group and we held counseling sessions for each of the participants who wanted to receive counseling.

**Table 1:** Contents of cognitive-behavior counseling sessions

Sessions	Contents of each session
Session 1	Participants’ introducing and getting familiar with each other and establishing the first contact, presenting the goals and rules of the group and the important issue of confidentiality, introducing methods and processes of treatment, explanation about and agreement on participatory nature of the sessions and necessity of doing homework, explanation and discussion about the meanings of self-esteem and body image and related factors
Session 2	Reviewing contents of the previous session, indicating the effects of cultural, social, and psychological pressures as well as life experiences and biography on self-esteem and body image, stating factors lowering or improving self-esteem, awareness of interactional nature of three systems, i.e. thinking, behavioral, and physiological systems, familiarity with Albert Ellis’ A-B-C model, practicing self-monitoring skills and presenting homework
Session3	Reviewing contents of previous sessions, checking homework, recognizing negative self-talks about body image and low self-esteem, practicing and improving positive self-talks about body image and self-worth, teaching deep breathing technique, teaching relaxation and practicing group relaxation with the participation of all members of the group, presenting homework
Session4	Reviewing contents of previous sessions, checking homework, analyzing the role of beliefs on body image and self-esteem, taking actions to inhibit maladaptive and negative thoughts and feelings through recognition of self-humiliating beliefs and replacing them with positive and adaptive thoughts, analyzing and challenging general fundamental misconceptions related to appearance and providing logical responses to them, practicing relaxation, presenting homework
Session 5	Reviewing contents of previous sessions, checking homework, reviewing vertical arrow, familiarity with advanced vertical arrow and types of beliefs, presenting homework
Session 6	Reviewing homework of previous sessions, discussing self-humiliating behaviors concerning body image and self-esteem, training techniques for changing troublesome behavior patterns, practicing relaxation, presenting homework
Session 7	Reviewing homework of previous sessions, giving the body its rights and having a good time, training pregnant women for having pleasant times with their bodies and improving their relationships with their bodies through creative involvement and specific exercises for enjoying and admiring their physical beings, training pregnant women for facing their probable physical defects, practicing relaxation as homework
Session 8	Reviewing homework and contents of previous sessions, presenting and reviewing techniques for protecting positive body image and self-esteem, reviewing taught strategies for changing negative beliefs, practicing relaxation, getting feedback about the program from participants and providing an opportunity to finish the group therapy program

Because of the COVID-19 pandemic and the need to maintain social distancing, the number of participants in each session was limited to at least three and no more than five people (depending on the available space in the health centers).

### Data instruments

This study’s measures included socio-demographic and obstetrics questionnaires, the Rosenberg self-esteem scale, the Multidimensional Body–Self Relations Questionnaire, and an exclusive breastfeeding questionnaire. Socio-demographic and obstetric questionnaires were completed prior to the study, and the Rosenberg self-esteem scale and Multidimensional Body–Self Relations Questionnaire were completed by both groups before the intervention, immediately after the completion of the intervention sessions, and then 4 weeks after childbirth. Similarly, one month after childbirth, a breastfeeding questionnaire was completed via interview.

### Socio-demographic and obstetrics questionnaire

The research team designed this questionnaire, which included questions about the participant’s and her spouse’s age, marriage duration, the participant’s and her spouse’s educational level and occupation, income level, marital satisfaction, the extent of spouse and family support, gestational age and fetus sex, history of pregnancy, childbirth and abortion, and successful breastfeeding history, and so on. Face and content validity were used to confirm the questionnaire’s validity.

### Rosenberg self-esteem questionnaire

The *Rosenberg self-esteem scale* was used to assess self-esteem (10 items). This questionnaire contains ten phrases, the first five of which are intended to be positive and the remaining five to be negative. The questionnaire is scored in reverse. This scale has a range of scores ranging from 0 to 30. Scores above 25 indicate high self-esteem, while scores between 15 and 25 indicate average self-esteem. Scores lower than 15 indicate low self-esteem. Cronbach’s alpha coefficients for this scale were calculated at 0.87 for men and 0.86 for women in the first turn and 0.88 for men and 0.87 for women in the second turn in the Makikangas study conducted in Finland [28] Cronbach’s alpha was reported to be 0.84 for the Iranian sample [41] Cronbach’s alpha was calculated to be 0.86 for the present study.

### Multidimensional body self-relations questionnaire

Cash et al. developed the *MBSRQ* in 1990, and its reliability was also confirmed. As a multidimensional measure of attitude toward body image, this questionnaire contains cognitive, emotional, and behavioral components, and its validity is reported to be 0.81. This questionnaire contains the following subscales: appearance evaluation, appearance orientation, fitness evaluation, fitness orientation, health evaluation, health orientation, illness orientation, body area satisfaction (BASS), self-classified weight, and overweight preoccupation. The scoring on this questionnaire is in reverse. Questions 6, 15, 16, 17, 23, 25, 28, 32, 33, 34, 37, 38, 40, 42, 43, 45, 47, 48, and 49 are scored in the opposite order. A person’s minimum score is 69, while a maximum score is 395. Higher scores indicate a higher level of physical satisfaction [29]. Rahati examined the validity and reliability of this questionnaire for Iranian samples in a study and found it to have a Cronbach’s alpha of 0.88 [31]. The present study’s Cronbach’s alpha was calculated to be 0.89.

### Exclusive breastfeeding questionnaire

The research team developed this questionnaire, which included questions about exclusive breastfeeding. Face and content validity were used to confirm the validity of this questionnaire.

### Sample size

G-power software was used to calculate the sample size, which was based on both self-esteem and body image variables. Based on a study conducted by Inanir et al. [42], with m1 = 118.3 (mean score of body image) and the assumption of a 25% increase in mean score due to intervention, m2 = 147.875, and power = 95%, a sample size of 32 people was calculated for each group, with a final sample size of 35 people in each group after accounting for 20% sample attrition. According to a study conducted by Barez et al. [32] and by considering m_1_ = 28.98 (mean score of self-esteem) and assuming a 25% increase in mean score due to intervention m_2_ = 34.776, and power = 95%, sample size was calculated 8 people in each group, and by considering the fact that sample size based on body image score was greater, the final sample size for each group was determined to be 35 people.

### Data analysis

Following data collection from all research units, the data was analyzed using SPSS 24. The K-S test confirmed the normality of the quantitative data. Chi-square, Chi-square for trend, independent-t, and Fisher’s exact tests were used to examine group homogeneity in terms of socio-demographic characteristics. Before the intervention, a t-test was used to compare mean scores on self-esteem and body image, as well as subscales of body image, among the groups; and repeated measures ANOVA with adjusting the baseline score and variables such as participants’ and their spouses’ educational level, participants’ age, and intervals between deliveries was used after intervention. Mauchly’s W was used to validate the repeated measures ANOVA. The Chi-square test was used to compare the frequency of exclusive breastfeeding among the groups. All of the tests were done with the intention-to-treat principle in mind.

## Results

**Fig. 1:**
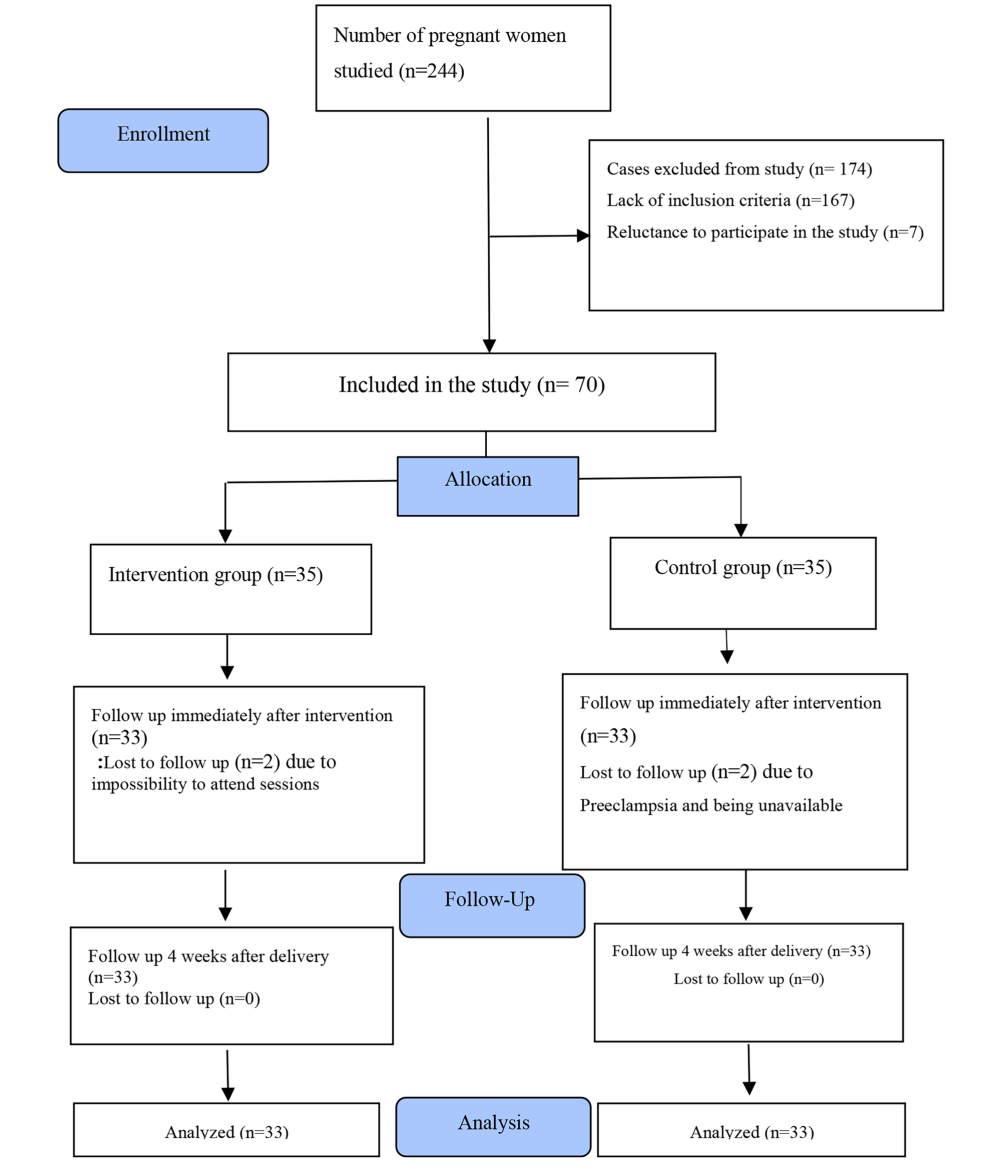
The study flow diagram

The study began in February 2020 but was halted for three months due to the COVID-19 pandemic. It was resumed in May 2020 and was completed in January 2021. In this study, two people from the control group (one due to preeclampsia and the other due to being unavailable) and two people from the intervention group (due to the COVID-19 pandemic and reluctance to attend the sessions on a regular basis) were excluded. Finally, 33 people from each group were studied (Fig. 1).

The mean (SD: standard deviation) ages of participants and their spouses in the intervention group were 30.51 (4.99) and 35.69 (5.02) years, respectively, and 27.89 (5.36) and 33.69 (5.86) years, respectively, in the control group. The intervention group had a mean (SD) gestational age of 26.86 (1.33) weeks and the control group had a gestational age of 26.91 (1.34) weeks. More than half of the intervention group participants (60%) and less than half of the control group participants (45.7%) had an academic education (p = 0.013). Table 2 shows the other socio-demographic characteristics of participants in both groups.

**Table 2:** Socio-demographic and obstetric characteristic among participants

Variable	Intervention group(n = 35) Number (percent)	Control group(n = 35) Number (percent)	p-value	variable	Intervention group (n = 35) Number(percent)	Control group (n = 35) Number (percent)	p-value
Age (years)*	30.51 (4.99)	27.89(5.36)	0.037	**Husband’s education** 0.026			
Husband’s age (years)*	35.69 (5.02)	33.69 (5.86)	0.130	Secondary school	0 (0)	(8.6) 3	
**Mother’s education**			0.013	High school	(2.9) 1	(2.9) 1	
Secondary school	0 (0)	(14.3) 5		Diploma	(31.4) 11	(45.7) 16	
High school	0 (0)	(8.6) 3		University	(65.7) 23	(42.9) 15	
Diploma	14 (40)	(31.4) 11		**Husband’s job** 0.458			
University	(60) 21	(45.7) 16		No job	0 (0)	(2.9) 1	
**Mother’s job** 0.259				Employee	(42.9) 15	(31.4) 11	
House work	(82.9) 29	(91.4) 32		Self-employee	(57.1) 20	(65.7) 23	
Employee	(17.1) 6	(5.7) 2		**Family support**0.821			
Self-employee	0 (0)	(2.9) 1		Very good	(45.7) 16	15 (42.9)	
**Family income**			1.000	Good	16 (45.7)	16 (45.7)	
Sufficient	12 (34.3)	10 (28.6)		Nearly good	2 (5.7)	4 (11.4)	
Insufficient	0 (0)	4 (11.4)		Not good	1 (2.9)	0 (0)	
Nearly sufficient	23 (65.7)	21 (60)		**Husband’s support** 0.259			
**Life satisfaction**			0.941	Very good	14 (40)	15 (42.9)	
Completely satisfied	17 (48.6)	19 (54.3)		Good	11 (31.4)	14 (40)	
Unsatisfied	4 (11.4)	4 (11.4)		Nearly good	6 (17.1)	6 (17.1)	
Relatively satisfied	14 (40)	12 934.3)		Not good	4 (11.4)	0 (0)	
**History of delivery** 0.811				**Planned pregnancy** 0.145			
Yes	17 (48.6)	16 (45.7)		Yes	25 (71.4)	30 (85.7)	
No	18 (51.4)	19 (54.3)		No	10 (28.6)	5 (14.3)	
**History of abortion** 0.771				**fetal sex** 0.632			
Yes	8 (22.9)	7 (20)		Female	19 (54.3)	17 (48.6)	
No	27 (77.1)	28 (80)		Male	16 (45.7)	18 (51.4)	
**History of successful breastfeeding** 0.245				**Wanted fetal sex** 1.000			
**Yes**	10 (29.4)	15 (42.9)		Yes	32 (91.4)	32 (91.4)	
**No**	24 (70.6)	20 (57.1		No	3 (8.6)	3 (8.6)	
Gestational age (week)*	26.91 (1.33)	26.85 (1.33)		Marriage duration* (years)	6.57 (4.23)	5.34 (3.42)	
Distance between deliveries * (years)	8.81 (2.42)	5.68 (2.98)	0.003	Previous B.F† duration*	17.57 (9.03)	23.20(5.18)	
**History of successful B.F†** 0.245				**Type of delivery** 0.228			
Yes	10 (29.4)	15 (42.9)		NVD	9 (27.3)	5 (15.2)	
No	24 (70.6)	20 (57.1)		CS	24 (72.7)	28 (84.8)	
**Exclusive B.F†** 0.007				**BMI‡ before pregnancy**	25.48 (4.20)	24.26(4.57)	
Yes	21 (63.6)	10 (30.3)		**before intervention BMI‡**	28.43 (4.43)	27.27(4.43)	
No	12 (36.4)	23 (69.7)		**after intervention BMI‡**	26.88 (3.86)	25.81(4.67)	

*these scores indicate the mean (SD)

The number of participants before intervention was 70 and after intervention was 66

†B.F: Breastfeeding ‡BMI: body mass index

Before the onset of counseling, the mean (SD) of self-esteem in the intervention group was 15.63 (3.18), while it was 17.83 (3.40) in the control group (p = 0.007). Self-esteem improved to 23.36 (2.34) in the intervention group and 18.40 (2.06) in the control group immediately following the intervention. Four weeks after childbirth, the mean self-esteem score in the intervention group was 24.36 (2.50) and 17.13 (3.40) in the control group. Prior to the intervention, an independent t-test revealed a significant statistical difference between the two groups (p = 0.007). Similarly, after the intervention, the intervention group’s mean self-esteem score was significantly higher than the control group, according to repeated measures ANOVA and after controlling for baseline score and variables such as participants’ and their spouses’ educational level, participants’ age, and intervals between their pregnancies (adjusted mean difference (AMD): 7.18; 95%confidence interval (CI): 4.43 to 9.94; p < 0.001) (Table 3).

**Table 3:** The mean (SD) of self-esteem before, immediately after intervention & 4 weeks after child birth

Variable	Intervention group n = 33 Mean (SD†)	Control group n = 33 Mean (SD)	AMD (95% CI) ‡	p-value
Before intervention	15.63 (3.18)	17.82 (3.39)	-2.20 (-3.77 to -0.63)	0.007
Immediately after intervention	23.36 (2.34)	18.40 (2.06)	7.18 (4.43 to 9.94)	< 0.001
4 weeks after childbirth	24.36 (2.50)	17.13 (3.40)		

The independent t-test was used for comparison of the groups before the intervention and repeated measure ANOVA with controlling baseline score and variables of participants & their spouse’s education level, participant’s age and interval between deliveries was used for comparing them after the intervention

The number of participants before intervention was 70 and after intervention was 66

†Standard Deviation

‡Adjusted Mean Difference (95% Confidence Interval)

Before the intervention, the intervention group’s mean (SD) body image was 214.48 (13.16) and the control group’s was 215.63 (14.10). This score was 264.86 (15.97) in the intervention group and 214.66 (21.67) in the control group immediately after the intervention, and it was 270.07 (15.25) in the intervention group and 211.00 (17.48) in the control group 4 weeks later. Prior to the intervention, the independent t-test revealed no statistically significant difference between the two groups (p = 0.727). However, after the intervention, the intervention group’s mean body image score was significantly higher than the control group, according to repeated measures ANOVA with the baseline score and variables such as participants’ and their spouses’ educational level, participants’ age, and intervals between their pregnancies (AMD: 49.74; 95%CI = 28.57 to 70.91; p < 0.001). The value of Mauchly’s W was 1 that indicates no departure from sphericity. The value of Partial Eta Squared in the “Sphericity Assumed” row was < 0.001 (Table 4).

**Table 4:** The mean (SD) of body image and its subscales before, immediately after and 4 weeks after childbirth

variable	Intervention group n = 33 Mean (SD†)	Control group n = 33 Mean (SD)	AMD ‡ (95%CI)	p-value
**Total body image**				
Before intervention	214.48 (13.16)	215.63 (14.10)	-1.14 (-7.65 to 5.36)	0.727
Immediately after intervention	264.86 (15.97)	214.67 (21.67)	49.74 (28.57 to70.91) <0.001	
4 weeks after delivery	270.07 (15.25)	211.0 (17.48)		
**Appearance evaluation**				
Before intervention	17.97 (2.87)	19.80 (4.30)	-1.83 (3.57 to -0.85)	0.041
Immediately after intervention	27.28 (4.60)	21.53 (4.88)	6.64 (1.80 to 11.48) 0.010	
4weeks after delivery	28.14 (4.90)	19.93 (3.08)		
**Appearance orientation**				
Before intervention	44.77 (6.34)	42.43 (5.15)	2.34 (-0.41 to 5.10)	0.094
Immediately after intervention	50.21 (3.62)	(6.44) 40.33	9.26 (4.71 to 13.82) 0 < 001	
4 weeks after delivery	50 (4.62)	41.66 (5.42)		
**Fitness evaluation**				
Before intervention	7.60 (1.63)	8.06 (1.89)	-0.46 (-1.30 to 0.38)	0.283
Immediately after intervention	11.14 (2.38)	(1.81) 7.53	3.30 (1.20 to 5.40) 0.004	
4 weeks after delivery	11.57 (2.47)	(1.60) 7		
**Fitness orientation**				
Before intervention	37.17(6.43)	38.03 (6.06)	-0.86 (-3.84 to 2.12)	0.568
Immediately after intervention	(6.17) 47.71	(6.63) 38.87	10.96 (5.06 to 16.85) 0.001	
4 weeks after delivery	(5.61) 49.28	(5.42) 35.07		
**Health evaluation**				
Before intervention	17.88 (2.16)	18.6 (2.81)	-0.71 (-1.91 to 0.48)	0.238
Immediately after intervention	23.57 (3.63)	18.27 (1.83)	5.73 (2.71 to 8.74) 0.001	
4 weeks after delivery	(2.82) 23.86	18.07 (2.63)		
**Health orientation**				
Before intervention	26.51 (2.92)	24.91 (3.54)	1.60 (0.05 to 3.15)	0.043
Immediately after intervention	32.28 (3.73)	25.47 (2.85)	4.53 (0.87 to 8.19) 0.018	
4 weeks after delivery	(2.84) 32.93	25.87 (3.77)		
**Illness orientation**				
Before intervention	18.71 (2.89)	18.31 (2.63)	0.40 (-0.92 to 1.72)	0.547
Immediately after intervention	20.71 (2.64)	17.47 (3.70)	4.37 (1.81 to 6.92) 0.002	
4 weeks after delivery	21.36 (2.24)	18.13 (2.47)		
**Pre BASS**				
Before intervention	25.23 (4.43)	26.71 (4.41)	-1.48 (-3.60 to 0.62)	0.165
Immediately after intervention	34.43 (5.62)	27.67 (6.18)	5.74 (-0.44 to 11.92) 0.067	
4 weeks after delivery	35.36 (5.34)	27.53 (5.19)		
**Self-classified weight**				
Before intervention	2.68 (0.98)	3.08 (1.02)	-0.40 (-0.88 to 0.08)	0.098
Immediately after intervention	2.68 (0.46)	2.9 (0.85)	-0.15 (-0.75 to 0.45) 0.594	
4 weeks after delivery	1.34 (0.23)	1.40 (0.48)		
**Overweight preoccupation**				
Before intervention	0.73 (0.10)	0.70 (0.12)	0.03 (-0.02 to 0.08)	0.217
Immediately after intervention	2.82 (0.61)	2.57 (0.47)	-0.18 (-0.77 to 0.40) 0.524	
4 weeks after delivery	2.82 (0.51)	2.65 (0.54)		

The independent t-test was used for comparison of the groups before the intervention and repeated measure ANOVA with controlling baseline score and variables of participants and their spouse’s education level, participant’s age & interval between deliveries was used for comparing them after the intervention

The number of participants was 70 before the intervention and 66 after the intervention

†standard deviation

‡mean difference (confidence interval 95%)

Body image has ten subscales, the mean score of which was significantly higher in the intervention group than the control group after intervention for appearance evaluation (p = 0.010), appearance orientation (p = 0.001), fitness evaluation (p = 0.004), fitness orientation (p = 0.001), health evaluation (p = 0.001), health orientation (p = 0.018), and illness orientation (p = 0.002) (Table 4).

According to the Chi-square test, 63.6% of mothers in the intervention group and 30.3% of mothers in the control group had exclusive breastfeeding, which was significantly higher in the intervention group than in the control group (p = 0.007).

## Discussion

The present study’s findings confirmed the efficacy of CBT in improving self-esteem and body image among breastfeeding women, resulting in an increase in the frequency of exclusive breastfeeding among women in the intervention group. Since there were no studies comparing the effectiveness of CBT on self-esteem and body image in other population groups, studies comparing the effectiveness of CBT on self-esteem and body image in other population groups were compared.

In the present study, cognitive-behavioral counseling improved self-esteem in women in the intervention group. In a study conducted by Vakilian et al. (2018) [27] to assess the efficacy of cognitive-behavioral counseling on the self-esteem of primiparous pregnant women, the efficacy of CBT in improving self-esteem scores was confirmed. The findings of a study conducted by Farahzadi et al. (2019) [33] to examine the effects of CBT on self-esteem in women suffering from body image dissatisfaction revealed that self-esteem improved significantly in the intervention group. The findings of Babadi’s study (2019) [34] on obese women with BMIs greater than 30 demonstrated the efficacy of CBT on self-esteem. However, in Myung-Sun Hyun’s study (2005) [43], self-esteem did not improve significantly in the CBT group. One of the reasons for this disparity could be the study’s different research population; Myung-sun Hyun’s study was conducted on adolescent boys. CBT can bring about emotional and behavioral changes, as well as improve self-esteem, by changing maladaptive thoughts and correcting fundamental misconceptions [44].

In the present study, cognitive-behavioral counseling improved body image and its domains in women in the intervention group. Similarly, in a study conducted by Navidian et al. (2016) [45] on women aged 20 to 40 three months after delivery, CBT resulted in improved body image in the intervention group compared to the control group. Similar to this study, the mean score of some subscales such as appearance evaluation and fitness evaluation was significantly higher in the intervention group than the control group. Ahmadi et al. (2017) [46] conducted a study on the effects of CBT on infertile women’s body image and concluded that CBT improved body image. CBT improved subscales of appearance evaluation, appearance orientation, and fitness evaluation in Ahmadi et al.‘s study, as it did in the present study. Furthermore, in their study, as in the present study, there was no significant difference in the self-classified weight between the two groups following the intervention. Appearance evaluation is a subscale selected on a global scale for assessing appearance concerns. This subscale evaluates the subject’s feelings about her physical attractiveness and satisfaction with her appearance [47]. The subscale appearance orientation investigates appearance-related biases and attitudes. The fitness evaluation also relates to the general fitness assessment and a self-classified weight evaluation examines the concern of the individual regarding his/her weight [45]. CBT was also effective in improving women’s body image after mastectomy in a study by Fadaei et al. (2011) [48]. By combining cognitive and behavioral approaches, cognitive-behavior therapy changes maladaptive thoughts and leads to a comprehensive understanding of body image and the factors that influence it [49].

The frequency of exclusive breastfeeding was significantly higher in the intervention group than in the control group in the present study. In Navidian’s study [45], the breastfeeding rate was also examined, and no significant difference between the two groups was discovered, which differed from the findings of the present study. The probable causes of this difference are cultural differences and different socioeconomic levels of the study populations in the two studies. Navidian’s research was carried out in one of the provinces with the lowest socioeconomic status. Furthermore, in our study, counseling sessions began during pregnancy and continued for 8 sessions; additionally, Navidian’s study was conducted only among primiparous women with no previous history of breastfeeding, whereas our study included both primiparous women and women with a second delivery.

In Sikander’s (2015) study [44], which was similar to ours, a cognitive-behavioral approach increased the frequency and length of exclusive breastfeeding; 59.6% of women in the intervention group had exclusive breastfeeding, whereas 28.6% of women in the control group had exclusive breastfeeding. The findings of Rahman’s (2011) study also demonstrate the efficacy of a cognitive-behavioral approach to exclusive breastfeeding [50]. Psychotherapy approaches create the cognitive environment required for behavior change. CBT, for example, gives mothers the opportunity to apply their knowledge in practice by presenting assignments and practices, which leads to long-term behavior change in mothers (Rahman et al., 2008) and, as a result, improvements in their health behaviors.

One of the study’s strengths was the one-month follow-up with participants after delivery. One of the study’s other strengths was the use of standard questionnaires. One of the study’s limitations was the low number of participants in counseling groups due to the pandemic Coronavirus. Furthermore, women with high-risk pregnancies or a history of depression were excluded from the study; thus, it is suggested to investigate the effects of CBT on women with high risk pregnancies in future studies. Furthermore, the follow-up time was only one month. However other variables such as the time of maternity leave and etc. may influence on the breastfeeding process that were not assessed in our study. Therefore, it is suggested that the future studies are conducted with longer follow-up as well as all possible confounders are considered.

## Conclusion

Counseling using a cognitive-behavioral approach improved self-esteem and body image in breastfeeding women, and as a result, the group that received counseling had a higher frequency of exclusive breastfeeding. Thus, it is suggested that women have access to self-esteem and body image counseling during their pregnancy; and because midwifery consultants are competent in various approaches of psychology as well as midwifery knowledge, their presence alongside mothers during the sensitive periods of pregnancy and breastfeeding can greatly benefit mothers and the health system.

## Data Availability

The datasets generated and/or analyzed during the current study are not publicly available due to limitations of ethical approval involving the patient data and anonymity but are available from the corresponding author on reasonable request.

## List of Abbreviations

CBT: Cognitive behavior therapy
IRCT: Iranian registry of the clinical trial
MBSRQ: Multidimensional body self-relations questionnaire
